# Potent In Vitro Activity of *Citrus aurantium* Essential Oil and *Vitis vinifera* Hydrolate Against Gut Yeast Isolates from Irritable Bowel Syndrome Patients—The Right Mix for Potential Therapeutic Use

**DOI:** 10.3390/nu12051329

**Published:** 2020-05-07

**Authors:** Maura Di Vito, Maria Grazia Bellardi, Maurizio Sanguinetti, Francesca Mondello, Antonietta Girolamo, Lorenzo Barbanti, Stefania Garzoli, Manuela Sabatino, Rino Ragno, Alberto Vitali, Ivana Palucci, Brunella Posteraro, Antonio Gasbarrini, Gian Maria Prati, Giovanni Aragona, Paola Mattarelli, Francesca Bugli

**Affiliations:** 1 Dipartimento di Scienze e Tecnologie Agro-Alimentari (DISTAL), Università di Bologna, Viale G. Fanin 42, 40127 Bologna, Italy; mariagrazia.bellardi@unibo.it (M.G.B.); lorenzo.barbanti@unibo.it (L.B.); paola.mattarelli@unibo.it (P.M.); 2 Dipartimento di Scienze Biotecnologiche di Base, Cliniche Intensivologiche e Perioperatorie, Università Cattolica del Sacro Cuore, Largo A. Gemelli 8, 00168 Rome, Italy; ivana_palucci1@yahoo.it (I.P.); francesca.bugli@unicatt.it (F.B.); 3 Dipartimento di Scienze di Laboratorio e Infettivologiche, Fondazione Policlinico Universitario A. Gemelli IRCCS, Largo A. Gemelli 8, 00168 Rome, Italy; 4 Dipartimento di Malattie Infettive, Istituto Superiore di Sanità, Viale Regina Elena 299, 00161 Rome, Italy; francesca.mondello@iss.it (F.M.); antonietta.girolamo@iss.it (A.G.); 5 Dipartimento di Chimica e Tecnologie del Farmaco, Università di Roma Sapienza, Piazzale Aldo Moro 5, 00100 Rome, Italy; stefania.garzoli@uniroma1.it; 6 Centro di Progettazione Molecolare di Roma—Dipartimento di Chimica e Tecnologie del Farmaco, Università di Roma Sapienza, Piazzale Aldo Moro 5, 00100 Rome, Italy; manuela.sabatino@uniroma1.it (M.S.); rino.ragno@uniroma1.it (R.R.); 7 Istituto di Scienze e Tecnologie Chimiche “Giulio Natta” SCITEC-CNR, Largo F. Vito 1, 00168 Rome, Italy; alberto.vitali@scitec.cnr.it; 8 Dipartimento di Scienze Gastroenterologiche, Endocrino-Metaboliche e Nefro-Urologiche, Fondazione Policlinico Universitario A. Gemelli IRCCS, Largo A. Gemelli 8, 00136 Rome, Italy; brunella.posteraro@unicatt.it; 9 Dipartimento di Scienze Biotecnologiche di Base, Cliniche Intensivologiche e Perioperatorie, Università Cattolica del Sacro Cuore, 00168 Rome, Italy; 10 Dipartimento di Scienze Mediche e Chirurgiche, Fondazione Policlinico Universitario A. Gemelli IRCCS, Largo A. Gemelli 8, 00136 Rome, Italy; antonio.gasbarrini@unicatt.it; 11 Dipartimento di Medicina e Chirurgia Traslazionale, Università Cattolica del Sacro Cuore, Largo A. Gemelli 8, 00168 Rome, Italy; 12 Dipartimento di Gastroenterologia e Epatologia, Ospedale “G. da Saliceto”, Via Taverna 49, 29121 Piacenza, Italy; gianmaria.prati@gmail.com (G.M.P.); g.aragona@ausl.pc.it (G.A.)

**Keywords:** *Candida species*, *Saccharomyces cerevisiae*, *Citrus aurantium* var. *amara* essential oil, *Vitis vinifera* cv Italia hydrolate, *Akkermansia muciniphila*

## Abstract

Background: Irritable bowel syndrome (IBS) is a functional disorder without any pathological alteration, in which the alterations of the *Candida*/*Saccharomyces* ratio of the gut microbiota, the balance of pro and anti-inflammatory cytokines and the brain-gut-microbiome axis are important for the development and progression of IBS. The aim of the study was to identify natural products, including essential oils or hydrolates, which were contextually harmless for the gut beneficial strains (e.g., *Saccharomyces* spp.) but inhibitory for the pathogenic ones (*Candida* spp.). Methods: The effectiveness of 6 essential oils and 2 hydrolates was evaluated using microbiological tests, carried out on 50 clinical isolates (*Candida*, *Saccharomyces* and *Galattomyces* species) and 9 probiotic strains (*Saccharomyces cerevisiae*, *Lactobacillus species*, *Akkermansia muciniphila* and *Faecalibacterium prausnitzii*) and immunological and antioxidant assays. Results: The study led to a mixture based on a 1/100 ratio of *Citrus aurantium* var. *amara* essential oil / *Vitis vinifera* cv Italia hydrolate able to contextually reduce, in a concentration-dependent manner, the ability of *Candida* species to form hyphal filaments and have an interesting immunomodulatory and anti-oxidant action. This mixture can potentially be useful in the IBS treatment promoting the restoration of the intestinal microbial and immunological balance.

## 1. Introduction

Irritable bowel syndrome (IBS) is a gastrointestinal disorder that affects around 11% of the population globally [1]. Despite being a chronic condition, contrary to the intestinal bowel disease (IBD) that is a chronic inflammation of the intestine, IBS is a functional disorder without any pathological alteration identified to date. However, IBS usually involves the brain-gut-microbiome axis but it can develop after an enteric infection, with the stress exacerbating the disorder-associated symptoms [2]. Symptoms reported in either IBS or IBD patients are almost the same but differ with respect to their extent. Generally, patients with IBD have more severe gastrointestinal symptoms compared with IBS. [3]. There is a growing evidence that the gut microbiota alteration (e.g., decrease in the relative abundance of a beneficial microorganism like *Akkermansia muciniphila*) and the psychology of the patient are both important for the development and progression of IBS [2]. Since 1985, authors have correlated the presence of *Candida* species in the intestinal tract with IBS [4] but only twenty years later other authors showed that yeasts are able to cause IBS symptoms in patients sensitized by *Candida* products, antigens and cross-antigens [5]. Other studies showed that the integration of *Saccharomyces cerevisiae* in the diet of patients with IBS was able to significantly reduce abdominal pain/discomfort and bloating and to improve the minimal clinically relevant threshold up to 10% [6,7,8]. In 2018, another study reported on the association between the relative abundance of *A. muciniphila* and the pain modulation in IBS patients [9]. Finally, studies showed that the concomitant decrease in the abundance of *Saccharomyces* species (i.e., *S. cerevisiae* and *S. cerevisiae* var. *boulardii*) and the increase of *Candida* species in the intestinal or vaginal microbial communities correlated with a decreased expression of the anti-inflammatory cytokine IL-10 and an increased expression of the pro-inflammatory cytokines IL-6, IL-8 and TNF-α [10,11,12,13].

Integrated treatments for IBS include the combined administration of conventional and complementary medications. The latter encompass Chinese medicine, homeopathy and phytotherapy [14,15]. Several studies have recently evaluated the efficacy of natural products, such as essential oils (EOs), in the treatment of IBS symptoms [16,17,18,19,20,21,22]. Some of the EOs studied derive from aromatic plants of the Mediterranean area, such as *Foeniculum vulgare*, *Pimpinella anisum*, *Mentha × piperita* and *Citrus aurantium*. The most active components of EOs are terpenes, whereas the hydrophilic hydrolates (Hys) are by-products of EO distillation (also known as hydrolates or aromatic waters) that contain up to 1% terpenic components of the EOs [23].

The aim of the present study was to evaluate the effectiveness of six EOs and two Hys, alone or in combination, on both yeast (*Candida*, *Saccharomyces* and *Galattomyces*) isolates from IBS patients and probiotic strains (*Saccharomyces* and *Lactobacillus*).

## 2. Materials and Methods 

### 2.1. EO and Hy Natural Substances

The six EOs studied were as follows—*Citrus lemon* var. *femminello* (femminello lemon), supplied by Exentiae Srl (Catania, Italy); *C. aurantium* var. *amara* (bitter orange), *Citrus lemon* (lemon) and *Citrus reticulata* (mandarin), supplied by Misitano and Stracuzzi Company (Messina, Italy); *Monarda didyma* (bee balm), supplied by the DISTAL of the Università di Bologna (Bologna, Italy) [24]; and *Cinnamomum zeylanicum* (cinnamon) supplied by APA-Ct (Forlì, Italy). All the *Citrus* species EOs were obtained from their respective fruit epicarps by pressing, whereas the *M. didyma* EO was obtained from the plant aerial parts and the *C. zeylanicum* EO was obtained from the cortex, both through steam distillation. The two Hys studied were from *M. didyma* and *Vitis vinifera* cv Italia, supplied by DISTAL of the Università di Bologna (Bologna, Italy). In particular, the *M. didyma* Hy was obtained from the same distillation process as for the *M. didyma* EO [25], whereas *V. vinifera*, for the first time in this study, was obtained according to the following protocol. Briefly, vegetal material (ripe berries and grape-stalks) of the *V. vinifera* cv Italia was collected from the plants grown in the Sicily region, monitored with a visual inspection to eliminate the parts apparently infested by bacteria and/or fungi and cut in small pieces that were subjected to distillation in a Clevenger-type apparatus. After one hour of distillation, only a sample of Hy (no EO was visualized) was collected.

### 2.2. Fungal and Bacterial Organisms

Fifty clinical isolates of fungal species (29 *Candida albicans*, 3 *Candida glabrata*, 1 *Candida krusei*, 4 *Candida parapsilosis*, 1 *Candida zeylanoides*, 8 *Galactomyces* species and 4 *S. cerevisiae*) were used, which had been recovered from fecal samples of IBS patients at the Dipartimento di Gastroenterologia e Epatologia, Ospedale G. da Saliceto of Piacenza, Italy. Patients were selected according to ROME III diagnostic criteria for IBS; all patients presented abdominal pain/discomfort for almost 3 days/weeks. The 62% of IBS patients had diarrhea (> 3 discharges/day) and the 38% suffered of constipation (<3 discharges/weeks). The Institutional Review Board of the Dipartimento di Gastroenterologia e Epatologia, Ospedale “G. da Saliceto” of Piacenza, Italy, approved the protocol for the obtainment of the patients’ fecal samples. Isolation and identification of fungal cultivable single-cell pure colonies from stool sample of the IBS patients were performed as previously described [24]. Two fluconazole-resistant *Candida tropicalis* isolates were also used, obtained from clinical stool samples of patients hospitalized at the Fondazione Policlinico Universitario A. Gemelli IRCCS of Rome, Italy, only to evaluate the effectiveness of natural products on the fungal morphology. For comparison purposes, nine probiotic strains were included, five non-commercial strains (*A. muciniphila* DSMZ 22959, *Faecalibacterium prausnitzii* DSMZ17677, *Lactobacillus acidophilus* LA3SACCO, *Lactobacillus plantarum* ATCC 14917, *Lactobacillus rhamnosus* DSMZ 20021) and four commercial strains (*S. boulardii* CBS5926, *L. acidophilus* LA-14, *Lactobacillus casei* RO215, *Lactobacillus helveticus* ROO52).

### 2.3. Broth Microdilution Susceptibility Testing

Broth microdilution (BMD) susceptibility tests was performed on all the 59 microbial organisms according to the European Committee on Antimicrobial Susceptibility Testing (EUCAST) international guidelines. RPMI broth (Irvine Scientific, St. Ana, CA, USA) was used for fungal organisms, Man, Rogosa and Sharpe (MRS) broth (Sigma Aldrich, St. Louis, MO, USA) for *Lactobacillus* species organisms and Brucella broth (Thermo Scientific, West Sussex, UK) for *A. muciniphila* and *F. prausnitzii* organisms. All the bacterial probiotic strains were allowed to grow in an anaerobic condition. Briefly, for each organism a cell suspension equal to 5 × 10^5^ cfu/mL was obtained and 50 μL of the suspension was dispensed in each well of a 96-well microtiter plate, which contained 50 μL of serial concentrations ranging from 4% *v*/*v* (40 mL/L) to 0.06% *v*/*v* (0.6 mL/L) for each EO and from 50% *v*/*v* (500 mL/L) to 1.67% *v*/*v* (16.7 mL/L) for each Hy. Tween 80 was utilized, at 0.05% *v*/*v*, as emulsifier for EO. Suitable positive controls, only with culture medium, were made for each microbiological strain. After 24/48 h of incubation at 37 °C, minimum inhibitory concentration (MIC) values were determined by spectrophotometric reading at 450 nm (EL808, Biotek, Winooski, VT, USA). To determine the minimum cytocidal concentration (MCC) or the minimum fungicidal concentration (MFC), 5 μL of the content of each well were seeded on standard medium agar plates, which were incubated at 37 °C for 24/48 h. For each natural substance (EO or Hy)/organism combination tested, the MIC was defined as the lowest concentration that allowed a complete inhibition of the organism’s growth compared with the growth in the substance-free control and the MCC as the lowest concentration that allowed the death of 99.9% or more of the initial inoculum organism. All tests were performed in triplicate. For *C. aurantium* var. *amara*, *C. zeylanicum* or *M. dydima* EOs, the BMD susceptibility tests were also performed with co-cultures of two randomly selected organisms (i.e., *C. albicans* clinical isolate 3.1 and *S. cerevisiae* clinical isolate 14.3), starting from a 5 × 10^5^ cfu/mL cell suspension obtained with a mixture (ratio 1:1) of the two isolates. Then, 10 μL of the content of each well showing the organism(s)’ visible growth was seeded on the Brilliance Candida agar chromogenic medium (Oxoid, Thermo Scientific, Basingstoke, UK) plates, which was incubated at 37 °C for 48 h. After incubation, the blue (*C. albicans*) and purple (*S. cerevisiae*) colonies were counted to obtain the cfu/mL values for each organism, respectively. Finally, to assess the effect of the *C. aurantium* var. *amara* EO and *V. vinifera* cv Italia combination, a checkerboard broth microdilution susceptibility testing was performed with the two cultured fungal organisms and sub-MIC concentration of *C. aurantium* var. amara EO (from 0.50% *v*/*v* to 0.06%) and *V. vinifera* cv Italia Hy (from 0.25% *v*/*v* to 0.06% *v*/*v*). Then, the growth percentage at each combination’s concentration was determined as the ratio between the number of cfu/mL obtained for that concentration and the number of cfu/mL obtained for the substance-free control. Finally, the content of each well was visualized directly with an inverted microscope and, after scraping of the bottom (50 μL), with an optical microscope to study the fungal morphology.

### 2.4. Peripheral Blood Mononuclear Cell (PBMC) Collection and ELISA Testing

Peripheral blood mononuclear cells (PBMCs) from human blood derived from refrigerated, banked healthy donor blood were collected with HISTOPAQUE-1077 (Sigma Aldrich, St. Louis, MO, USA) solution according to the manufacturer’s protocol. After collection, 250 μL of a suspension of 1.2 × 10^6^ cells/mL were cultured in sextuplicate in a 48-well cell culture plate at 37 °C overnight. PBMCs were incubated in the presence of serial dilutions of the *V. vinifera* Hy (from 50% *v*/*v* to 6.25% *v*/*v*) alone or in combination with *C. aurantium* var. *amara* EO (from 0.50% *v*/*v* to 0.06% *v*/*v*) or with lipopolysaccharide (LPS, 1 μg/mL). Untreated PBMC cells were used as control. After 24 h of incubation at 37 °C, the content of 4 replicates was mixed and centrifuged (400 × g for 10 min at room temperature) to separate the cells from the culture medium, which was stored at −20 °C until testing. The remaining two replicates were treated with 5 μL of Alamar Blue reagent (Invitrogen, Carlsbad, CA, USA) in order to study the cellular vitality. To evaluate the expression of IL-6, IL-8, TNF-α and IL-10 cytokines, ELISA tests (AVIVA Systems, San Diego, CA, USA) were performed using 100 μL of the aforementioned culture medium, according to the manufacturer’s instructions. In each well, the content of cytokines was evaluated with optical reading using a microplate reading set to 450 nm. A calibration curve with six serial dilutions of the standard was included in each experiment, which was repeated three times.

### 2.5. DPPH (2,2-Diphenyl-1-Picryl-Hydrazyl-Hydrate) Assay

The DPPH antioxidant assay was performed to evaluate the scavenging activity of the *V. vinifera* Hy. Serial dilutions of the Hy (100 μL) were mixed with the 400-μM DPPH solution (100 μL) in a 96-well microtiter plate. Methanol was used as a negative control. The mixtures were incubated for 30 min at 37 °C in the dark and the change in spectrophotometric absorbance at 517 nm was measured. Mean values were obtained from triplicate experiments. The percentage of radical scavenging activity per sample was calculated using the following equation:

% inhibition = [(A DPPH − A Extract)/ADPPH] × 100,

where A DPPH is the absorbance of the DPPH solution only and A Extract is the absorbance of the tested sample. The IC50 value for the *V. vinifera* Hy was defined as the concentration of organic compound at which 50% of DPPH radicals was quenched and was expressed as mean ± standard deviation.

### 2.6. GC-MS Analysis

A *V. vinifera* cv Italia Hy sample (50 mL) was extracted with diethyl ether (100 mL). The collected organic layers dried over dry Na2SO4 and deprived of the solvent at reduced pressure furnished about 21.5 mg of an oily mixture (0.043%). The resulting Hy extract (1 mg) as well as a *C. aurantium* var. *amara* EO sample (1 mg) were separately diluted in 1mL methanol and used for Gas Chromatography-Mass Spectrometry (GC/MS) analysis. A GC-MS Perkin Elmer Clarus 500 instrument equipped with flame ionization detector (FID) and a Restek Stabilwax fused-silica capillary column (length 60 m × 0.25 mm ID) was employed. Helium was used as the carrier gas with a flow rate of 1 mL/min and the oven temperature program was as follows—5 min at 60 °C then a gradient of 5 °C/minute to 220 °C. One µL of sample was manually injected at 280 °C into the Gas Chromatography injector in the splitless mode. Relative percentages for quantification of the components were calculated by electronic integration of the GC-FID peak areas. Identification of the constituents was performed on MS library search (Wiley and Nist). Linear retention indices (LRIs) of each compound were calculated using a mixture of aliphatic hydrocarbons (C8-C30, Ultrasci) injected directly into GC injector.

### 2.7. Cytotoxicity Assays

Human gingival fibroblasts were obtained as described from by Radicioni G. et al. [26]. Cells were grown in Dulbecco’s Modified Eagle Medium (DMEM) supplemented with 10% fetal calf serum (FCS) and were not used beyond the fifth step. The cytotoxicity of serial dilutions of the mixture of *C. aurantium* var. *amara* EO (from 0.50% *v*/*v* to 0.03% *v*/*v*) and *V. vinifera* cv Italia Hy (from 50% *v*/*v* to 3.25% *v*/*v*) for 24 and 48 h was evaluated. Untreated culture cells were used as control. Tetrazolium compound [3-(4,5-dimethylthiazol-2-yl)-5-(3-carboxymethoxyphenyl)-2-(4-sulfophenyl)-2H-tetrazolium, inner salt; MTS] (Promega, Madison, WI, USA) was used, which was diluted in basic medium. Then, 1 × 10^4^ cells were suspended in 200 mL of basic medium to be coated in 96-well cell culture plates and grown at confluence for 24 h. Cell vitality was evaluated after the addition of the aforementioned serial dilutions at 37 °C for 24 and 48 h. The MTS analysis was performed according to the manufacturer’s protocols. Each experiment was done in triplicate.

### 2.8. Statistical Analysis

All data obtained in triplicate from each experiment were expressed as means ± standard deviation (SD). The response of *S. cerevisiae* and *C. albicans* to *C. aurantium* var *amara* EO concentration was fitted by means of mathematical models, using the Sigma Plot 10 (Systat Software Inc., Chicago, IL, USA) software. The best fitting model was chosen between linear and exponential decay function. The two respective equations are:

y = y0 + ax(1)

y = a × exp(−bx)(2)

where: y0, intercept; a, slope (Equation (1)), initial level (Equation (2)); b, rate constant.

All the results obtained from the broth microdilution susceptibility testing were presented as relative values, assuming the average of *S. cerevisiae* and *C. albicans* as positive control (Ctrl+) = 100%. The data were treated with the GraphPad V 4.0 software. A two-way ANOVA for the factors clinical isolates (2 levels) and EO-Hy combined treatments (13 levels including Ctrl+) was run on these data. The CoStat 6.3 package (CoHort Software, Berkeley, CA, USA) was used for the ANOVA.

All the results, obtained by cytotoxicity study, were expressed as mean percentage of vitality and data treated with GraphPad V 4.0 software. The treated group means were compared by analysis of variance (ANOVA) followed by a multiple comparison of means using the Dunnett’s test. Values with *p* < 0.05 were considered significant.

## 3. Results

### 3.1. Antimicrobial Activities of EOs

Table 1 shows the MIC values of the 38 *Candida* species isolated from IBS patients against the EOs of *C. lemon*, *C. lemon* var. *femminello*, *C. reticulata*, *C. aurantium* var. *amara, C. zeylanicum* and *M. didyma* tested at the 0.50% *v*/*v* to 0.06% *v*/*v* concentrations. 

The MIC50 values were > 0.50% *v*/*v* for *C. lemon*, *C. lemon* var. *femminello* and *C. reticulata*, 0.50% *v*/*v* for *C. aurantium* var. *amara*, ≤ 0.06% *v*/*v* for *C. zeylanicum* and 0.25% *v*/*v* for *M. didyma*. 

Table 2 shows the MIC values of the 7 probiotic strains and the 12 *Galactomyces* species and *S. cerevisiae* isolates against the EOs of *C. aurantium* var. *amara*, *C. zeylanicum* and *M. didyma* (the only three EOs showing better activity) tested at the 4.0% *v*/*v* to 0.06% *v*/*v* concentrations. It was chosen to test lower EO concentrations for the isolates from pathogenic species (i.e., *Candida* species) and higher EO concentrations for the isolates from beneficial species (i.e., probiotic or *Galactomyces*/*S. cerevisiae* species). This choice would ensure the activity toward the pathogenic species and non-toxicity toward the beneficial species eventually present in the gut microbiota of IBS patients. The MIC50 values were > 4.0% *v*/*v* for *C. aurantium* var. *amara*, 0.50% *v*/*v* for *M. didyma* and ≤ 0.06% *v*/*v* for *C. zeylanicum.*

**Figure 1:**
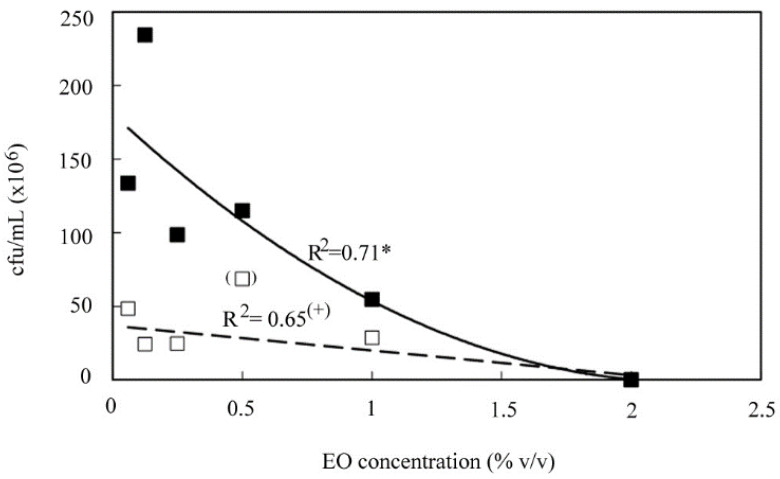
cfu/ml of *Saccharomyces cerevisiae* (filled symbol) and *Candida albicans* (empty symbol) in response to *Citrus aurantium* var. *amara* essential oil (EO) concentration. Solid line, exponential decay function (Equation (2)) describing *S. cerevisiae* abatement at increasing EO concentration. Dashed line, linear function (Equation (1)) describing *C. albicans* abatement at increasing EO concentration. ^(+)^ and *, significant at *p* ≤ 0.10 and *p* ≤ 0.05, respectively.

Based on these findings, the *C. aurantium* var. *amara*, *C. zeylanicum* and *M. didyma* EOs were tested against two co-cultured clinical isolates of *C. albicans* (clinical isolate 3.1) and *S. cerevisiae* (clinical isolate 14.3), which were representative of both pathogenic and beneficial isolates included in the study. Using 4% *v*/*v* to 0.015% *v*/*v* concentrations, it was found that MIC and MFC values were equal for all the three EOs. Expectedly, the *C. zeylanicum* EO was the most effective (MFCs for *C. albicans* and *S. cerevisiae*, 0.015% *v*/*v* and 0.06% *v*/*v*, respectively), compared to the *M. didyma* EO (MFCs for *C. albicans* and *S. cerevisiae*, 1% *v*/*v*) and the *C. aurantium* var. *amara* EO (MFCs for *C. albicans* and *S. cerevisiae*, 2% *v*/*v*). Therefore, the inhibitory capability of the *C. aurantium* var. *amara* EO was further investigated by counting the cfu/mL of both *C. albicans* and *S. cerevisiae* recovered after their incubation with sub-MIC concentrations (≤ 2% *v*/*v*) of the EO. Figure 1 shows that the 2% concentration of *C. aurantium* var. *amara* EO was able to completely suppress both organisms. Interestingly, *S. cerevisiae* exhibited an exponential trend of increased tolerance to the EO (R^2^ = 0.71) that was statistically significant and different (*p* < 0.05) from the linear trend of *C. albicans* (R^2^ = 0.65).

As expected, neither *M. dydima* nor *V. vinifera* cv Italia Hys showed antimicrobial activities against all the 59 microbial strains above mentioned.

### 3.2. Immunomodulatory and Antioxidant Activities of the V. vinifera cv Italia Hy

The immunomodulatory activities of both *V. vinifera* cv Italia and *M. didyma* Hys were studied, using human PBMCs. Data showed that PBMCs treated with *M. didyma* Hy died whereas those treated with *V. vinifera* cv Italia Hy did not lose viability. Therefore, the effects of the *V. vinifera* cv Italia Hy and, in parallel, LPS alone or in combination with the Hy has been investigated, on the levels of IL-10 and TNF-α produced by PBMCs. 

**Figure 2:**
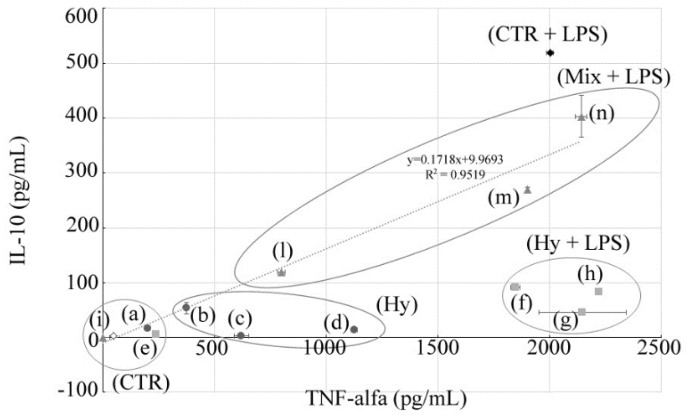
Variation of the amount of cytokines IL-10 and TNF-α in the medium culture of PBMCs treated with scalar dilutions of the *V. vinifera* cv Italia Hy alone or in combination with EO and/or LPS. The circle-shaped indicators refer to the samples treated with the Hy only; the squares are referred to the samples treated with the Hy plus LPS, while the triangles show the samples treated with Hy plus the EO of *C. aurantium* var. *amara* plus LPS. The white rhombus is the untreated control and the black rhombus is the control treated with LPS. Letters **a**, **b**, **c** and **d** indicate samples treated with scalar dilution of the Hy (50% *v*/*v*, 25% *v*/*v*, 12.50% *v*/*v* and 0.06% *v*/*v* respectively). Letters **e**, **f**, **g** and **h** are referred to samples treated with scalar dilution of the Hy (50% *v*/*v*, 25% *v*/*v*, 12.50% *v*/*v* and 0.06% *v*/*v* respectively) and LPS (1 μgr/mL). Letters **i**, **l**, **m** and **n** are referred to PBMCs treated with a 1:100 *v*/*v* mixture of the Hy, (50% *v*/*v*, 25% *v*/*v*, 12.50% *v*/*v* and 0.06% *v*/*v* respectively) and the EO (0.50% *v*/*v*, 0.25% *v*/*v*, 0.12% *v*/*v* and 0.06% *v*/*v* respectively) plus LPS.

Table 3 summarizes both the mean and standard deviation values of IL-10 and TNF-α under the tested conditions, while Figure 2 shows the relationship of the two cytokines in each condition. 

As shown in Table 3, the levels of IL-10 and TNF-α in untreated PBMCs were 2.6 ± 0.07 pg/mL and 47.98 ± 2.8 pg/mL, respectively, whereas those in PBMCs treated with LPS were 518 pg/mL ± 3.0 pg/mL and 2002.9 pg/mL ±14.4 pg/mL. The IL-10 and TNF-α values of PBMCs treated with 50% of the *V. vinifera* cv Italia Hy and those of PBMCs treated with both 50% of the *V. vinifera* cv Italia Hy and LPS had values close to those of the untreated PBMCs (Figure 2, letters a and e in CTR circle). The values of PBMCs treated with concentrations other than 50% of *V. vinifera* cv Italia Hy alone varied based on the Hy concentration tested. In particular, only TNF-α values were higher than those of PBMCs, while both IL-10 and TNF-α values were lower than those of PBMCs stimulated with LPS alone (Figure 2, letters b, c, d in Hy circle). PBMCs treated with concentrations other than 50% of *V. vinifera* cv Italia Hy in combination with LPS had a strong reduction of IL-10 values, compared to PBMCs stimulated with LPS only.

The DPPH assay was used to study the anti-oxidant activity of the *V. vinifera* cv Italia Hy. Data show that the Hy exhibited low activity because of an IC50 of 4 ×10-3 ± 0.001 μg/μL of organic component, which corresponds to the one present in a 95.6 ± 2.1% *v*/*v* of Hy solution (i.e., to the one of the almost pure Hy). 

### 3.3. Effectiveness of the C. aurantium var. Amara EO and V. vinifera cv Italia Hy Combination

**Figure 3:**
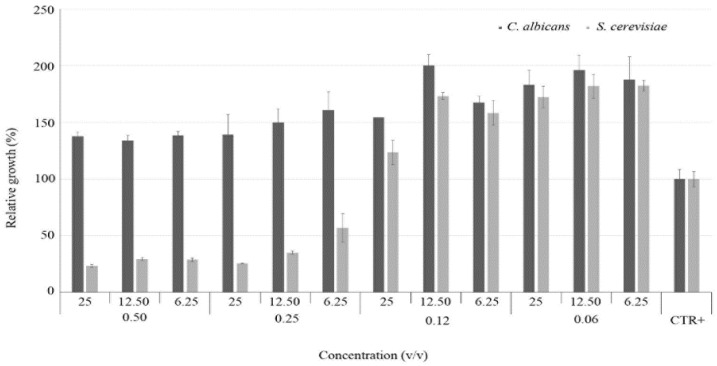
Relative growth of the clinical isolates of *C. albicans* (3.1) and S. cerevisiae (14.3) when cultured in presence of scalar dilutions of both the EO of *C. aurantium* var *amara* and the Hy of *V. vinifera* cv Italia. The values are relative to the positive control (CTR+) and are expressed in percentage. The average of the two isolates’ positive controls is set at 100%.

Figure 3 shows the results of checkerboard BMD testing of *C. aurantium* var. *amara* EO (from 0.50% *v*/*v* to 0.06% *v*/*v*) and *V. vinifera* cv Italia Hy (from 25% *v*/*v* to 6.25% *v*/*v*) against *C. albicans* (clinical isolate 3.1) and *S. cerevisiae* (clinical isolate 14.3). As showed by ANOVA test, concentrations equal to or lower than 0.12% *v*/*v* for EO, regardless of Hy concentration, did not produce any statistically significant inhibition for the two isolates, whereas for concentrations of the EO higher than 0.12% *v*/*v* the isolate of *S. cerevisiae* shows a statistically significant growth inhibition. 

Then, 1:100 *v*/*v* mixtures of EO (0.50% *v*/*v* to 0.06% *v*/*v*) and Hy (50% *v*/*v* to 6.25%) were evaluated for morphological microscopy examination and five of seven *Candida* species (exceptions were one *C. glabrata* and one *C. tropicalis* [0.4 R]) isolates displayed a concentration-dependent reduced capability of forming hyphal filaments (Table 4). As expected, any morphological alterations for the *S. cerevisiae* isolate tested were observed. Additionally, none of the EO/Hy mixture concentrations was able to affect the nine probiotic isolates as well the *A. muciniphila* and *F. prausnitzii* isolates (potentially usable as probiotics).

It was assessed whether the EO/Hy mixture concentrations had immunomodulatory activities on human PBMCs when used in combination with LPS. 

As shown in Figure 2, a linear decrease of IL-10 and TNF-α values were observed in PBMCs treated with both LPS and different concentrations of the EO/Hy mixture. In particular, at the lowest concentration tested (6.25% *v*/*v* Hy and 0.625% *v*/*v* EO) the values of cytokines are similar to those expressed by the PBMC stimulated with LPS only. Whereas, by increasing the tested concentrations of the EO/Hy mixture, the IL-10 and TNF-α values decrease linearly (Figure 2, letters l, m and n in Mix + LPS circle) until they become similar to those of the PBMCs (Figure 2, letter i in CTR circle). 

**Figure 4:**
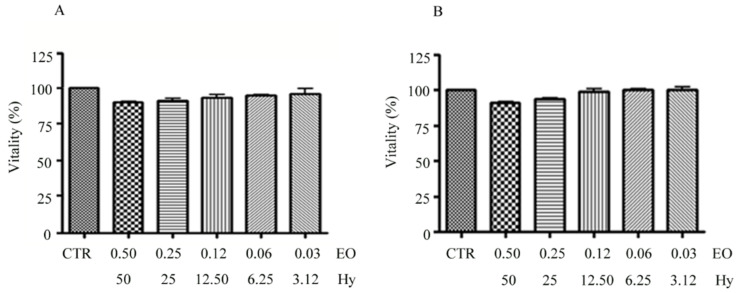
Cytotoxicity tests. Cytotoxicity assays performed with a Cell Titer Blue viability assay on human gingival fibroblasts at 24 (**A**) and 48 h (**B**) upon cell synchronization (24 h). Cells were treated with scalar dilution of a 1:100 *v*/*v* mixtures of *Citrus aurantium* var. *amara* EO (0.50% *v*/*v* to 0.06% *v*/*v*) and *Vitis vinifera* cv Italia Hy (50% *v*/*v* to 6.25%).

Furthermore, it was investigated whether the EO/Hy mixture concentrations had cytotoxic activities on human gingival fibroblasts. As shown in Figure 4, none of the concentrations tested was capable of producing statistically significant effects on the fibroblasts after 24 h and 48 h of incubation.

Finally, GC-MS analyses performed on both the EO and the organic fraction from Hy showed that the major component of EO was the limonene (93.93%) and the major components of the Hy were limonene (17.68%), cis-geraniol (25.40%) and β-linalool (38.81%) (Table 5).

## 4. Discussion

Few studies have been done on the effectiveness of essential oils in treating patients with IBS and most of them have been made in the last 10 years. In addition to the now known properties of the essential oil of *Mentha × piperita* that make it an interesting treatment not only for IBD but also for IBS [22], other essential oils, especially those obtained from aromatic plants with known carminative activity, have been recently studied in the treatment of IBS. Mahboubi M. [18] reviewed the action of *Zataria multiflora* EOs in the treatment of gastrointestinal problems, especially in IBS. As reported, 60 drops oral daily dose of *Z. multiflora* essential oil (2%) can relieve the symptoms of IBS without any adverse effects. Portincasa P. et al. [20] and Di Ciaula A. et al. [16] investigated the activity of the EOs of *F. vulgaris* in association with *Curcuma longa* extract in IBS patients. The two studies concluded that the administration of these natural compounds significantly improved symptoms and quality of life in IBS patients within 2 months from the treatment. Mosaffa-Jahromi M. et al. [19] have demonstrated that a treatment of 4 weeks with encapsulated *P.anisum* EO (200 mg for 3 times a day) is able to improve IBS symptoms better than placebo or *M. piperita* capsules (Colpermin^®^) (*p* < 0.0001). Finally, Agah S. et al. [27] studied the effect of 20 drops of *Cuminum cyminum* EO on 57 patients with IBS selected according to the ROME II diagnostic criteria. Data showed that IBS symptoms had a statistically significant reduction during and after treatment.

According to the most recent literature, at the base of the IBS there is an alteration of the intestinal microbiota in favor of microbial strains generally present in lower percentages (especially fungal strains). For this reason, we chose to investigate the antimicrobial action of some EOs and Hys on both fungal and probiotic strains. All the natural products studied were obtained from plants potentially cultivable in the Mediterranean area with the exception of *C. zeylanicum*, which was taken as a reference given its known high antimicrobial activity [28]. Furthermore, considering that the IBS symptoms are deeply conditioned by an altered brain-gut-microbiome axis, some of the EOs in study have been chosen to potentially act not only on microbial strains but also on the central nervous system (CNS). Therefore, 4 out of the 6 EOs studied belong to the *Citrus* genus which, besides being a genus widely present in Italy, is mainly used in aromatherapy in the treatment of nervous disorders and anxiety. Particularly, the *C. aurantium* var. *amara* EO is used as a natural mild sedative, hypnotic and anticonvulsant agent. It is used in treating insomnia, sleep disorders, anxiety, epilepsy and seizures. It has been also reported for its anti-spasmodic effects and, thanks to the presence of limonene, for its gastro-protective and ulcer healing actions by increasing the gastric production of mucus, which is useful as a secondary intervention in the treatment of chronic inflammatory diseases. Similarly, *C. limon* and *C. reticulata* EOs are used in phytotherapy to relieve symptoms of anxiety [29]. Instead, the *M. didyma* is an aromatic plant included in the BELFRIT (Belgium, France and Italy) list, native of Northern Europe but cultivable in Italy; its EO has two phenols as major components (thymol and carvacrol) and therefore shows a marked antimicrobial activity [24].

The study of MICs (and MCCs), against clinical isolates of *Candida* spp., indicates that no EOs belonging to the *Citrus* genus have an action comparable to that of *M. didyma* (MIC50 = 0.25% *v*/*v*) and *C. zeylanicum* (MIC50 ≤ 0.06 % *v*/*v*) EOs. All the EOs obtained from *Citrus* have a MIC50 > 0.5 % *v*/*v* with the exception of the EO of *C. aurantium* var. *amara* that showed a MIC50 = 0.50% *v*/*v*, slightly different from that of *M. didyma*. Based on this result, the action of the 3 EOs with major antifungal activity has been tested towards both probiotic strains and beneficial clinical isolates belonging to the intestinal microbiota (Table 2). The data indicate that both the above are generally more resistant to the action of EOs than the pathogenic fungal isolates. In particular, the EO of *C. aurantium* var. *amara* has a MIC50 value (MIC50 ≥ 4% *v*/*v*) at least 3 dilutions higher against the beneficial clinical isolates than against the clinical isolates of *Candida* species (MIC50 = 0.50% *v*/*v*).

These data are in agreement with those available in the literature highlighting that the probiotic strains have higher MIC values than the pathogenic strains [3,30,31] and that thanks to the different MIC values between probiotic and pathogenic strains it is possible to administer probiotics and EOs together to cure pathogenic infections in human gut [32].

To evaluate the effectiveness of the three best EOs on the rebalance of beneficial and pathological clinical strains, we performed the BMD susceptibility tests using the EOs of *C. aurantium* var. *amara*, *M. dydima* and *C. zeylanicum* against two co-cultured randomly selected fungal clinical isolates (*C. albicans* clinical isolate 3.1 and *S. cerevisiae* clinical isolate 14.3).

The choice to co-culture one clinical isolate of *C. albicans* with one of *S. cerevisiae* is motivated by the evidence that in IBS patients a greater colonization of fungal strains belonging to the genus *Candida*, at the expense of the ones of *S. cerevisiae*, has been demonstrated [4,5,33]. If on the one hand it is known that *S. cerevisiae* is a yeast generally well represented in the intestinal microbiota of healthy subjects that, besides having a known anti-inflammatory action, is able to compete for the territory with *Candida* spp., on the other we know that IBS patients are characterized by a higher abundance of *C. albicans* (61.8%) compared to healthy subjects (48.75%) [33]. Moreover, IBS *C. albicans* isolates were different from those of healthy subjects not only genotypically but also phenotypically as these clinical isolates of *C. albicans* were able to grow at high temperatures and low pH compared to those isolated from healthy subjects [33]. These data suggest that IBS could be characterized by the presence of potentially virulent *C. albicans* strains and motivate our choice.

Our data show that, when co-cultured, the two fungi have the same MFC but, by analyzing the fungal growth in the presence of sub-MIC of *C. aurantium* var. *amara* EO, it is possible to identify a different modulating action of this EO towards the two clinical strains. In particular, for concentrations equal to or lower than 2% *v*/*v* of *C. aurantium* var. *amara* EO, the strain of *C. albicans* is significantly more inhibited than that of *S. cerevisiae* (Figure 1). Therefore, this activity could be useful in an attempt to rebalance the relative abundance of each clinical species in IBS patients.

The variation of the intestinal microbiota also involves the variation of the intestinal inflammatory microenvironment. Indeed, it is known that the decrease in *S. cerevisiae* and the increase in *Candida* species is generally related to an increase in the pro-inflammatory pattern in IBS patients [5]. As described in Results, an interesting immunomodulatory and anti-oxidant property of the *V. vinifera* cv Italia Hy was identified; on the contrary, the Hy of *M. dydima* was excluded because toxic for human PBMCs culture.

The *V. vinifera* is a plant genus recognized by both traditional and consolidated use for the treatment of microcirculation problems thanks to its important anti-oxidant, anti-inflammatory, spasmolytic and immunomodulated actions. The first sources on the use of *V. vinifera* as medicament are the Egyptian papyri, the Sumerian tablets, the writings of Hippocrates of Cos, Celsus, Galen and Paracelsus. Currently, natural products obtained from *V. vinifera* are found on the market in the form of dry extracts or soft aqueous extracts [34,35,36] but it is hard to find the hydrolate of *V. vinifera* because it is an unusual natural product obtained from the distillation of a non-aromatic plant. This means that our data are innovative showing that it is obtained from a still unexplored natural product that, thanks to its immunomodulatory and anti-oxidant activity, could be exploited in the chronic low-grade inflammation that establishes in patients with IBS [37].

Tests developed to identify the best combination of *C. aurantium* var. *amara* EO and *V. vinifera* cv Italia Hy have shown that a serial dilution of a mixture made with a 1:100 ratio of the two natural products (starting from a concentration of 0.50% *v*/*v* of *C. aurantium* var. *amara* EO and 50% *v*/*v* of *V. vinifera* cv Italia Hy) caused an interesting inhibition of the ability of *C. albicans* species to form hyphae (Table 4). As reported in a recent review, the morphological plasticity of *Candida albicans* cells makes this fungus able to adapt to the different niches present in the gut of the host organisms. Generally, the production of *C. albicans* hyphae and pseudo-hyphae is typical of cellular models of virulence specialized in the commensalism [38]. The above are in agreement with what has been observed on *C. albicans* clinical isolates of IBS patients [33] where the gut microbiota is characterized by a greater abundance of *C. albicans* cells, more virulent than in healthy subjects. In this context, the use of one of the effective serial dilutions based on *C. aurantium* var. *amara* EO and *V. vinifera* cv Italia Hy, able to inhibit the production of hyphae and pseudo-hyphae of *C. albicans* strains, could prove to be successful in IBS treatment; especially considering that the same serial dilution of the two natural compounds also has the ability to linearly inhibit in a concentration-dependent manner the production of the IL-10 and TNF-α cytokines from PBMCs grown in presence of the pro-inflammatory stimulus (LPS) (Figure 2). Rather than the use of intestinal cells such as CaCo2, the ex vivo experiments carried out on PBMC were performed in order to evaluate the action of natural compounds in modulating the human systemic immunological response. This latter activity would enhance the effectiveness of the mixture inducing the reduction of the pro-inflammatory framework generally present in patients and able to favoring the growth of *Candida* species and promoting the establishment of the appropriate microenvironment for the development of beneficial probiotic strains including *A. muciniphila* and *F. prausnitzii* recently proposed as markers of the syndrome [39].

Finally, the mixture identified in this study appears to be suitable for an oral use in patients with IBS because, in addition to showing no in vitro cytotoxic activity (Figure 4), it appeared to be composed mainly of the monoterpene limonene because all the other chemical constituents were present in traces (Table 5). In a study, the limonene has been designated as a compound with low oral toxicity based on its lethal dose (LD50) and repeated-dose toxicity studies. Therefore, except for topical applications, for which attention must be paid to avoid its irritating potential, the use of limonene is widely granted in the pharmaceutical, cosmetic and food industries for its many properties [40].

## 5. Conclusions

In conclusion, even though further in vivo studies to evaluate the effectiveness as well as the possible presence of adverse and/or toxic effects are necessary, our study concludes that a mixture based on *C. aurantium* var. *amara* EO and *V. vinifera* cv Italia Hy (ratio 1:100) can be useful in the treatment of IBS. The use of this mixture in an integrated treatment with conventional treatments could favor the growth of both probiotic and beneficial gut strains, disfavoring pathogenic fungal strains and rebalancing the intestinal inflammatory microenvironment through the reduction of the expression of IL-10 and TNF-α cytokines and, in parallel, of the pro-inflammatory microenvironment.

## Figures and Tables

**Table 1:** MIC values of selected clinical *Candida* spp. isolates against the essential oils in study.

Designation	Isolates	MIC (% *v*/*v*)					
		CL^1^	CL-f^2^	CR^3^	CA-a^4^	CZ^5^	MD^6^
3.1	*C. albicans*	>0.50	>0.50	>0.50	>0.50	≤0.06	0.25
3.2	*C. albicans*	>0.50	>0.50	>0.50	0.50	≤0.06	0.25
3.3	*C. albicans*	>0.50	>0.50	>0.50	0.50	≤0.06	0.25
3.4	*C. albicans*	>0.50	>0.50	>0.50	0.50	≤0.06	0.25
3.5	*C. albicans*	>0.50	>0.50	>0.50	0.50	≤0.06	0.25
3.6	*C. albicans*	>0.50	>0.50	>0.50	0.06	≤0.06	0.25
3.7	*Pikia kudriavzeii*	>0.50	>0.50	>0.50	0.25	≤0.06	0.25
3.8	*C. albicans*	>0.50	>0.50	>0.50	0.50	≤0.06	0.25
3.9	*C. albicans*	>0.50	>0.50	>0.50	0.50	≤0.06	0.25
3.11	*C. albicans*	>0.50	>0.50	>0.50	0.50	≤0.06	0.25
3.12	*C. albicans*	>0.50	>0.50	>0.50	0.50	≤0.06	0.25
3.14	*C. albicans*	>0.50	>0.50	>0.50	0.50	≤0.06	0.25
3.15	*C. albicans*	>0.50	>0.50	>0.50	>0.50	≤0.06	0.25
3.16	*C. albicans*	>0.50	>0.50	>0.50	0.50	≤0.06	0.25
3.17	*C. albicans*	>0.50	>0.50	>0.50	0.50	≤0.06	0.25
3.18	*C. albicans*	>0.50	>0.50	>0.50	0.50	≤0.06	0.25
3.19	*C. albicans*	>0.50	>0.50	>0.50	0.50	≤0.06	0.50
5.2	*C. parapsilosis*	>0.50	>0.50	>0.50	0.50	≤0.06	0.12
5.3	*C. zeylanoides*	>0.50	>0.50	>0.50	0.50	≤0.06	0.12
5.4	*C. parapsilosis*	>0.50	>0.50	>0.50	>0.50	≤0.06	0.25
5.5	*C. parapsilosis*	>0.50	>0.50	>0.50	>0.5	≤0.06	0.25
5.6	*C. parapsilosis*	>0.50	>0.50	>0.50	>0.50	≤0.06	0.25
8.1	*C. albicans*	>0.50	>0.50	>0.50	>0.50	≤0.06	0.25
9.1	*C. albicans*	>0.50	>0.50	>0.50	>0.50	≤0.06	0.25
10.1	*C. albicans*	>0.50	>0.50	>0.50	>0.50	≤0.06	0.50
10.2	*C. albicans*	>0.50	>0.50	>0.50	>0.50	≤0.06	0.50
10.3	*C. albicans*	>0.50	>0.50	>0.50	>0.50	≤0.06	0.25
10.4	*C. albicans*	>0.50	>0.50	>0.50	>0.50	≤0.06	0.25
10.5	*C. albicans*	>0.50	>0.50	>0.50	>0.50	≤0.06	0.25
10.6	*C. albicans*	>0.50	>0.50	>0.50	>0.50	≤0.06	0.25
10.7	*C. albicans*	>0.50	>0.50	>0.50	>0.50	≤0.06	0.25
10.8	*C. albicans*	>0.50	>0.50	>0.50	>0.50	≤0.06	0.25
11.1	*C. albicans*	>0.50	>0.50	>0.50	>0.50	≤0.06	0.25
11.2	*C. albicans*	>0.50	>0.50	>0.50	>0.50	≤0.06	0.25
14.1	*C. albicans*	>0.50	>0.50	>0.50	>0.50	≤0.06	0.25
18.2	*C. glabrata*	>0.50	>0.50	>0.50	>0.50	≤0.06	0.50
18.5	*C. glabrata*	>0.50	>0.50	>0.50	>0.50	≤0.06	0.50
18.6	*C. glabrata*	>0.50	>0.50	>0.50	>0.50	≤0.06	0.50

MIC values were found to be equal to the MFC values for all the isolates tested. ^1^*C. limon*. ^2^*C. limon* var. *femminello*. ^3^*C. reticulata*. ^4^*C. aurantium* var. *amara*. ^5^*C. zeylanicum*. ^6^*M. dydima.*

**Table 2:** MIC values of commercial probiotic strains and clinical beneficial bacterial strains against the essential oils of *M. dydima, C. aurantium* var. *amara*, *C. zeylanicum*.

**Strain**	**Probiotic Commercial Strains**	**MIC (% *v*/*v*)**		
		**CA-a ^1^**	**CZ ^2^**	**MD ^3^**
**CBS5926**	*S. boulardii*	>4.00	≤0.06	0.50
LA3SACCO	*L. acidophilus*	>4.00	≤0.06	2.00
LA-14	*L. acidophilus*	>4.00	≤0.06	0.50
ATCC14917	*L. plantarum*	>4.00	≤0.06	1.00
DSMZ 20021	*L. rhamnosus*	>4.00	≤0.06	0.50
R0215	*L. casei*	>4.00	≤0.06	1.00
R0052	*L. helveticus*	0.12	≤0.06	0.12
**Designation**	**Beneficial Clinical Strains**			
13.0	*Galactomyces* species	2.00	≤0.06	0.25
17.1	*Galactomyces* species	>4.00	≤0.06	2.00
17.5	*Galactomyces* species	4.00	≤0.06	0.50
17.7	*Galactomyces* species	>4.00	≤0.06	0.50
17.8	*Galactomyces* species	>4.00	≤0.06	0,50
17.9	*Galactomyces* species	>4.00	≤0.06	0.50
17.10	*Galactomyces* species	4.00	≤0.06	0.50
17.11	*Galactomyces* species	4.00	≤0.06	1.00
14.3	*S. cerevisiae*	1.00	≤0.06	0.25
14.5	*S. cerevisiae*	1.00	≤0.06	0.25
14.9	*S. cerevisiae*	1.00	≤0.06	0.50
14.11	*S. cerevisiae*	2.00	≤0.06	0.25

MIC values were found to be equal to the MFC values for all the isolates tested. ^1^ *C. aurantium* var. *amara*, ^2^ *C. zeylanicum,* ^3^ *M. dydima*.

**Table 3:** IL-10 and TNF-α values of both peripheral blood mononuclear cells (PBMCs) and PBMCs stimulated with lipopolysaccharide (LPS) when treated with the Hy of *V. vinifera* alone or in combination with the EO of *C. aurantium* var. *amara*.

Simple	VV-Hy^1^	CA-EO^2^	Mean (± St. Dev.)^3^	
	(%*v*/*v*)	(%*v*/*v*)	IL-10	TNF-α
**PBMCs**	-	-	2.60 (± 0.07)	47.98 (± 2.80)
	50.00	-	16.40 (± 0.80)	199.50 (± 12.30)
	25.00	-	53.80 (± 10.20)	375.80 (± 7.20)
	12.50	-	14.51 (± 0.60)	1126.80 (± 14.80)
	6.25	-	3.28 (± 0.30)	620.60 (± 33.20)
**PBMCs + LPS**	-	-	518.00 (± 3.00)	2002.90 (±14.40)
	50.00	-	5.93 (± 0.30)	238.70 (± 7.50)
	25.00	-	90.90 (± 3.60)	1846.30 (± 20.30)
	12.50	-	83.70 (± 4.20)	2221.50 (± 2.60)
	6.25	-	46.30 (± 0.90)	2148.20 (± 195.6)
	50.00	0.5	0.64 (± 0.14)	0.64 (± 0.14)
	25.00	0.25	119.00 (± 0.46)	977.91 (± 16.60)
	12.50	0.12	269.53 (± 4.07)	1901.90 (± 0.00)
	6.25	0.06	403.23 (± 37.65)	2143.01 (± 25.09)

^1^ hydrolate of *V. vinifera* cv Italia, ^2^Essential oil of *C. aurantium* var. *amara*, ^3^values are expressed as pg/mL.

**Table 4:** Effectiveness of a mixture of *C. aurantium* var. *amara* EO and *V. vinifera* cv italia Hy on the morphology of clinical fungal species.

Designation	Fungal Species	*C. aurantium* va. *amara* (% *v*/*v*) EO + *V. vinifera* Hy					
		0.50 + 50	0.25 + 25	0.12 + 12.5	0.06 + 6.25	0.03 + 3.17	CTR
0.3R ^1^	*C. tropicalis*	Y	H	H	H	H	H++
0.4R ^1^	*C. tropicalis*	H++ (no adhesion)	H++ (no adhesion)	H++ (no adhesion)	H++ (no adhesion)	H++ (no adhesion)	H++
3.1	*C. albicans*	Y	H	H+	H+	H+	+
10.1	*C. albicans*	Y	H	H+	H+	H++	H++
3.15	*C. albicans*	Y	Y	Y	Y	H+	H++
8.5	*C. glabrata*	Y	Y	Y	Y	Y	Y
3.7	*C. krusei*	Y	Y	Y	Y	Y	Y
5.1	*C. parapsilosis*	Y	Y	Y	Y	Y	Y

^1^ fluconazole resistant strain, Y =Presence of a lot of yeast; H = Presence of rare hyphae; H+ = few hyphae and pseudo-hyphae, H++ = a lot of hyphae and pseudo-hyphae.

**Table 5:** Chemical composition (%) of *C. aurantium* var. *amara* EO and *V. vinifera* Hy.

Name ^1^	LRI ^2^	LRIlit ^3^	%CA-EO ^4^	%VV-Hy ^5^
α-pinene	1038	1040	0.57	0.39
β-pinene	1134	1130	0.73	-
sabinene	1152	1156	0.36	-
β-myrcene	1171	1167	2.37	-
limonene	1232	1235	93.93	17.68
γ-terpinene	1270	1263	0.26	-
o-cymene	1295	1287	0.15	-
cis-linaloloxide	1433	1420	-	0.85
decanal	1520	1515	0.22	-
β-linalol	1553	1547	0.21	38.81
linalyl acetate	1572	1575	0.77	-
α-terpineol	1705	1700	0.22	7.55
borneol	1724	1717	-	0.69
cis-geraniol	1831	1823	-	25.40
humulene-1,2-epoxide	2018	NA*	-	2.55
d-nerolidol	2030	2023	0.21	-
thymol	2198	2189	-	1.75
carvacrol	2231	2225	-	4.33
SUM			100.00	100.00

^1^ Elution order on polar column; ^2^ Linear Retention indices measured on polar column; ^3^ Linear Retention indices from literature; ^4^ Essential oil of *C. aurantium* var. *amara*, ^5^ hydrolate of *V. vinifera* cv Italia, * Normal Alkane RI. The %VV-Hy are referred to the fraction of organic components extracted from 50 mL of Hy.

## References

[1] Canavan C., West J., Card T. (2014). The epidemiology of irritable bowel syndrome. Clin. Epidemiol..

[2] Abdul Rani R., Raja Ali R.A., Lee Y.Y. (2016). Irritable bowel syndrome and inflammatory bowel disease overlap syndrome: Pieces of the puzzle are falling into place. Intest. Res..

[3] Lee A.D., Spiegel B.M., Hays R.D., Melmed G.Y., Bolus R., Khanna D., Khanna P.P., Chang L. (2017). Gastrointestinal symptom severity in irritable bowel syndrome, inflammatory bowel disease and the general population. Neurogastroenterol. Motil..

[4] Petitpierre M., Gumowski P., Girard J.P. (1985). Irritable bowel syndrome and hypersensitivity to food. Ann. Allergy.

[5] Santelmann H., Howard J.M. (2005). Yeast metabolic products, yeast antigens and yeasts as possible triggers for irritable bowel syndrome. Eur. J. Gastroenterol. Hepatol..

[6] Cayzeele-Decherf A., Pélerin F., Leuillet S., Douillard B., Housez B., Cazaubiel M., Jacobson G.K., Jüsten P., Desreumaux P. (2017). *Saccharomyces cerevisiae* CNCM I-3856 in irritable bowel syndrome: An individual subject meta-analysis. World J. Gastroenterol..

[7] Spiller R., Pélerin F., Cayzeele Decherf A., Maudet C., Housez B., Cazaubiel M., Jüsten P. (2016). Randomized double blind placebo-controlled trial of *Saccharomyces cerevisiae* CNCM I-3856 in irritable bowel syndrome: Improvement in abdominal pain and bloating in those with predominant constipation. United Eur. Gastroenterol. J..

[8] Choi C.H., Jo S.Y., Park H.J., Chang S.K., Byeon J.S., Myung S.J. (2011). A randomized, double-blind, placebo-controlled multicenter trial of *Saccharomyces boulardii* in irritable bowel syndrome: Effect on quality of life. J. Clin. Gastroenterol..

[9] Cruz-Aguliar R.M., Wantia N., Clavel T., Vehreschild M.J.G.T., Buch T., Bajbouj M., Haller D., Busch D., Schmid R.M., Stein-Thoeringer C.K. (2018). An open-labeled study on fecal microbiota transfer in irritable bowel syndrome patients reveals improvement in abdominal pain associated with the relative abundance of *Akkermansia Muciniphila*. Digestion.

[10] Sokol H., Leducq V., Aschard H., Pham H.P., Jegou S., Landman C., Cohen D., Liguori G., Bourrier A., Nion-Larmurier I. (2017). Fungal microbiota dysbiosis in IBD. Gut.

[11] Di Vito M., Fracchiolla G., Mattarelli P., Modesto M., Tamburro A., Padula F., Agatensi L., Giorlandino F.R., Girolamo A., Carbonara G.G. (2016). Probiotic and Tea Tree Oil treatments improve therapy of vaginal candidiasis: A preliminary clinical study. Med. J. Obstet. Gynecol..

[12] Bashashati M., Rezaei N., Shafieyoun A., McKernan D.P., Chang L., Öhman L., Quigley E.M., Schmulson M., Sharkey K.A., Simrén M. (2014). Cytokine imbalance in irritable bowel syndrome: A systematic review and meta-analysis. Neurogastroenterol. Motil..

[13] Bennet S.M., Polster A., Törnblom H., Isaksson S., Capronnier S., Tessier A., Le Nevé B., Simrén M., Öhman L. (2016). Global cytokine profiles and association with clinical characteristics in patients with irritable bowel syndrome. Am. Gastroenterol..

[14] Farhadi A., Bruninga K., Fields J., Keshavarzian A. (2001). Irritable bowel syndrome: An update on therapeutic modalities. Expert Opin. Investig. Drugs.

[15] Peckham E.J., Nelson E.A., Greenhalgh J., Cooper K., Roberts E.R., Agrawal A. (2013). Homeopathy for treatment of irritable bowel syndrome. Cochrane Database Syst. Rev..

[16] Di Ciaula A., Portincasa P., Maes N., Albert A. (2018). Efficacy of bio-optimized extracts of turmeric and essential fennel oil on the quality of life in patients with irritable bowel syndrome. Ann. Gastroenterol..

[17] Botschuijver S., Roeselers G., Levin E., Jonkers D.M., Welting O., Heinsbroek S.E.M., de Weerd H.H., Boekhout T., Fornai M., Masclee A.A. (2017). Intestinal fungal dysbiosis is associated with visceral hypersensitivity in patients with irritable bowel syndrome and rats. Gastroenterology.

[18] Mahboubi M. (2019). Therapeutic Potential of *Zataria multiflora* Boiss in Treatment of Irritable Bowel Syndrome (IBS). J. Diet. Suppl..

[19] Mosaffa-Jahromi M., Lankarani K.B., Pasalar M., Afsharypuor S., Tamaddon A.M. (2016). Efficacy and safety of enteric coated capsules of anise oil to treat irritable bowel syndrome. J. Ethnopharmacol..

[20] Portincasa P., Bonfrate L., Scribano M.L., Kohn A., Caporaso N., Festi D., Campanale M.C., Di Rienzo T., Guarino M., Taddia M. (2016). curcumin and fennel essential oil improve symptoms and quality of life in patients with Irritable Bowel Syndrome. A. J. Gastrointestin. Liver Dis..

[21] Thompson A., Meah D., Ahmed N., Conniff-Jenkins R., Chileshe E., Phillips C.O., Claypole T.C., Forman D.W., Row P.E. (2013). Comparison of the antibacterial activity of essential oils and extracts of medicinal and culinary herbs to investigate potential new treatments for irritable bowel syndrome. BMC Complement. Altern. Med..

[22] Alam M.S., Roy P.K., Miah A.R., Mollick S.H., Khan M.R., Mahmud M.C., Khatun S. (2013). Efficacy of Peppermint oil in diarrhea predominant IBS—A double blind randomized placebo—Controlled study. Mymensingh Med. J..

[23] D’Amato S., Serio A., Chaves Lopez C., Paparella A. (2018). Hydrosols: Biological activity and potential as antimicrobials for food applications. Food Control.

[24] Strati F., Di Paola M., Stefanini I., Albanese D., Rizzetto L., Lionetti P., Calabrò A., Jousson O., Donati C., Cavalieri D. (2016). Age and gender affect the composition of fungal population of the human gastrointestinal tract. Front. Microbiol..

[25] Mattarelli P., Epifano F., Minardi P., Di Vito M., Modesto M., Barbanti L., Bellardi M.G. (2017). Chemical composition and antimicrobial activity of essential oils from aerial parts of *Monarda didyma* and *Monarda fistulosa* cultivated in Italy. J. Essent. Oil Bear. Plants.

[26] Radicioni G., Stringaro A., Molinari A., Nocca G., Longhi R., Pirolli D., Scarano E., Iavarone F., Manconi B., Cabras T. (2015). Characterization of the cell penetrating properties of a human salivary proline-rich peptide. Biochim. Biophys. Acta.

[27] Agah S., Taleb A.M., Moeini R., Gorji N., Nikbakht H. (2013). Cumin extract for symptom control in patients with irritable bowel syndrome: A case series. Middle East J. Dig. Dis..

[28] Committee on Herbal Medicinal Products (HMPC) (2011). Assessment Report on Cinnamomum Verum J.S. Presl, Cortex and Corticis Aetheroleum.

[29] Dosoky N.S., Setzer W.N. (2018). Biological activities and safety of *Citrus* spp. essential oils. Int. J. Mol. Sci..

[30] Di Vito M., Modesto M., Girolamo A., Ballardini M., Tamburro A., Meledandri M., Mondello F. (2015). *In vitro* activity of tea tree oil vaginal suppositories against *Candida* spp. and probiotic vaginal microbiota. Phytother. Res..

[31] Petretto G.L., Fancello F., Zara S., Foddai M., Mangia N.P., Sanna M.L., Omer E.A., Menghini L., Chessa M., Pintore G. (2014). Antimicrobial activity against beneficial microorganisms and chemical composition of essential oil of *Mentha suaveolens* ssp. insularis grown in Sardinia. J. Food Sci..

[32] Shipradeep Karmakar S., Sahay Khare R., Ojha S., Kundu K., Kundu S. (2012). Development of probiotic candidate in combination with essential oils from medicinal plant and their effect on enteric pathogens: A review. Gastroenterol. Res. Pract..

[33] Sciavilla P., Strati F., Prati G.M., Fornari F., Modesto M., Luiselli D., Cavalieri D., De Filippo C., Mattarelli P. (2016). Metagenomic characterisation of gut microbiota in IBS patients. UEG WEEK (United European Gastroenterology Week). United Eur. Gastroent..

[34] Miller S.A., White J.A., Chowdhury R., Gales D.N., Tameru B., Tiwari A.K., Samuel T. (2018). Effects of consumption of whole grape powder on basal NF-κB signaling and inflammatory cytokine secretion in a mouse model of inflammation. J. Nutr. Intermed. Metab..

[35] Terra X., Pallarés V., Ardèvol A., Bladé C., Fernández-Larrea J., Pujadas G., Salvadó J., Arola L., Blay M. (2011). Modulatory effect of grape-seed procyanidins on local and systemic inflammation in diet-induced obesity rats. J. Nutr. Biochem..

[36] Committee on Herbal Medicinal Products (HMPC) (2017). Assessment Report on Vitis vinifera L., folium.

[37] Ohman L., Simrén M. (2010). Pathogenesis of IBS: Role of inflammation, immunity and neuroimmune interactions. Nat. Rev. Gastroenterol. Hepatol..

[38] Noble S.M., Gianetti B.A., Witchley J.N. (2017). *Candida albicans* cell-type switching and functional plasticity in the mammalian host. Nat. Rev. Microbiol..

[39] Lopez-Siles M., Enrich-Capó N., Aldeguer X., Sabat-Mir M., Duncan S.H., Garcia-Gil L.J., Martinez-Medina M. (2018). Alterations in the abundance and co-occurrence of *Akkermansia muciniphila* and *Faecalibacterium prausnitzii* in the colonic mucosa of inflammatory bowel disease subjects. Front. Cell. Infect. Microbiol..

[40] Kim Y.W., Kim M.J., Chung B.Y., Bang du Y., Lim S.K., Choi S.M., Lim D.S., Cho M.C., Yoon K., Kim H.S. (2013). Safety evaluation and risk assessment of d-Limonene. J. Toxicol. Environ. Health B Crit. Rev..

